# The Effects of Altitude Training on Erythropoietic Response and Hematological Variables in Adult Athletes: A Narrative Review

**DOI:** 10.3389/fphys.2018.00375

**Published:** 2018-04-11

**Authors:** Kamila Płoszczyca, Józef Langfort, Miłosz Czuba

**Affiliations:** ^1^Department of Sports Training, Academy of Physical Education of Katowice, Katowice, Poland; ^2^Department of Physiology, Institute of Sport, Warsaw, Poland

**Keywords:** erythropoietin, altitude training, hypoxia, blood oxygen capacity, hematological variables

## Abstract

**Background:** One of the goals of altitude training is to increase blood oxygen-carrying capacity in order to improve sea-level endurance performance in athletes. The elevated erythropoietin (EPO) production in hypoxia is a key factor in the achievement of enhanced hematological variables. The level of the EPO increase and acceleration of erythropoiesis depend on the duration of exposure and degree of hypoxia. Furthermore, many other factors may affect the hematological response to altitude training.

**Aim:** The purpose of this narrative review was to: (1) analyze the kinetics of EPO and hematological variables during and after altitude training; (2) summarize the current state of knowledge about the possible causes of individual or cohort differences in EPO and hematological response to altitude training; (3) formulate practical guidelines for athletes to improve the efficiency of altitude training.

**Methods:** A narrative review was performed following an electronic search of the databases PubMed/MEDLINE and SPORTDiscus via EBSCO for all English-language articles published between 1997 and 2017.

**Results:** Complete unification of results from studies on EPO kinetics was difficult due to different time and frequency of blood sampling by different researchers during and after altitude training, but the data presented in the reviewed literature allowed us to detect certain trends. The results of the reviewed studies were divergent and indicated either increase or no change of hematological variables following altitude training. Factors that may affect the hematological response to altitude training include hypoxic dose, training content, training background of athletes, and/or individual variability of EPO production.

**Conclusions:** Despite the potential benefits arising from altitude training, its effectiveness in improving hematological variables is still debatable. Further research and better understanding of factors influencing the response to altitude, as well as factors affecting the suitable measurement and interpretation of study results, are needed.

## Introduction

Nowadays, altitude training has become a standard training protocol in many aerobic sports to increase exercise capacity at sea level or to acclimatize prior to competitions at altitude or before ascending to altitude (Wilber, 2007). Sudden exposure of the human body to a hypoxic environment or staying at altitude induces numerous adaptations which can lead to improved athletes' performance at sea level. These mechanisms are generally attributed to either hematological (Levine and Stray-Gundersen, 1992), cardiovascular (Naeije, 2010), or ventilatory (Townsend et al., 2016) effects of altitude training. However, altitude training can also lead to improved muscle buffering capacity (Gore et al., 2001), increased glycolytic enzyme activity (Katayama et al., 2004), enhanced capillary density (Vogt et al., 2001), muscle mitochondrial volume (Geiser et al., 2001), and myoglobin concentration (Terrados et al., 1990; Zoll et al., 2006). Expected effects may be achieved by applying one of the recognized high-altitude training methods, i.e., live high-train high (LH-TH), live high-train low (LH-TL), and live low-train high (LL-TH). An up-to-date survey of altitude training methods with a modified nomenclature which analyzed the opportunities to combine different hypoxic methods was conducted by Millet et al. (2013).

In the classical LH-TH method, which is the first concept of altitude training, the athletes live and train at moderate altitudes (2,000–3,000 m) to stimulate erythropoiesis, which increases erythrocyte volume, thus enhancing sea-level endurance performance. This method is still in use today, but one major conclusion drawn from scientific research and sport practice is that athletes are not able to train at an equivalent, or near-equivalent, intensity as at sea level. Thus, athletes may return to sea level in a detrained state (Wilber, 2007). To overcome this problem, athletes often descend from altitude to perform intensive training sessions, returning to altitude at night to continue the acclimatization process. This LH-TL model of altitude training was developed in the early 1990s (Levine and Stray-Gundersen, 1992) and received considerable attention. Today, several training strategies can be used based on the LH-TL principles. Athletes can live in a natural hypobaric hypoxic environment, or use special technologies such as nitrogen dilution or oxygen filtration to create a normobaric hypoxic environment (Mattila and Rusko, 1996; Gore et al., 2001). However, there are still many controversies about the mechanisms of hematological and non-hematological adaptations to LH-TL and the extent of enhanced sport performance (Lundby and Robach, 2016). The quality and quantity of the results in the literature are still insufficient to elucidate the mechanism of the effect of LH-TL on sport performance. It is considered that the positive effects of LH-TL are mainly due to the increase of red cell volume (RCV) (Levine and Stray-Gundersen, 2005), but there exists a contrary opinion that improvements in the energy cost of exercise seem more likely than increases in RCV as a result of LH-TL (Gore and Hopkins, 2005).

In the LL-TH protocol, athletes live under normoxic conditions and train in a natural, hypobaric, or simulated normobaric hypoxic environment. The LL-TH method can be used by athletes at rest (intermittent hypoxic exposure; IHE) or during training sessions (intermittent hypoxic training; IHT) (Terrados et al., 1988; Casas et al., 2000; Czuba et al., 2011). However, during the LL-TH protocol, the exposure to acute hypoxia is too short (1–2 h per day), and insufficient to modify hematological variables (Czuba et al., 2011, 2017, 2018), but the LL-TH method can contribute to the activation of non-hematological adaptive mechanisms (Girard et al., 2017; Millet and Girard, 2017).

Although the concept of altitude or hypoxic training to improve sea-level sport performance has been known for nearly 50 years, its efficacy remains somewhat controversial. Whilst some studies support the ergogenic effects of altitude training on sport performance (Mattila and Rusko, 1996; Gore et al., 2001), others do not (Ashenden et al., 2000; Hinckson et al., 2005). There are also conflicting reports on the efficacy of altitude training in improving hematological variables. These discrepancies may be due to differences in duration of hypoxia exposure, intensity of the hypoxic stimulus, type of training model, volume and intensity of exercise during the experiment, and the sports skill level of study participants. Methodological approaches and measurement techniques used by researchers are also significant.

Therefore, the aim of this narrative review was to focus on kinetics of EPO and hematological variables during and following altitude training, and to review the current state of knowledge about the possible causes of the differences in EPO response and hematological response to altitude/hypoxic training. Furthermore, short practical recommendations for athletes were formulated for improving efficacy of altitude training.

## Methods

An electronic search was performed using the databases PubMed/MEDLINE and SPORTDiscus via EBSCO for all English-language articles in the fields of medicine, the health professions, biochemistry, genetics and molecular biology published between 1997 and 2017. Keyword searches included combinations of the following terms: [“erythropoietin” OR “epo”] AND [“altitude training” OR “hypoxic training” OR “hypoxia”] AND [“athletes”].

**Figure 1:**
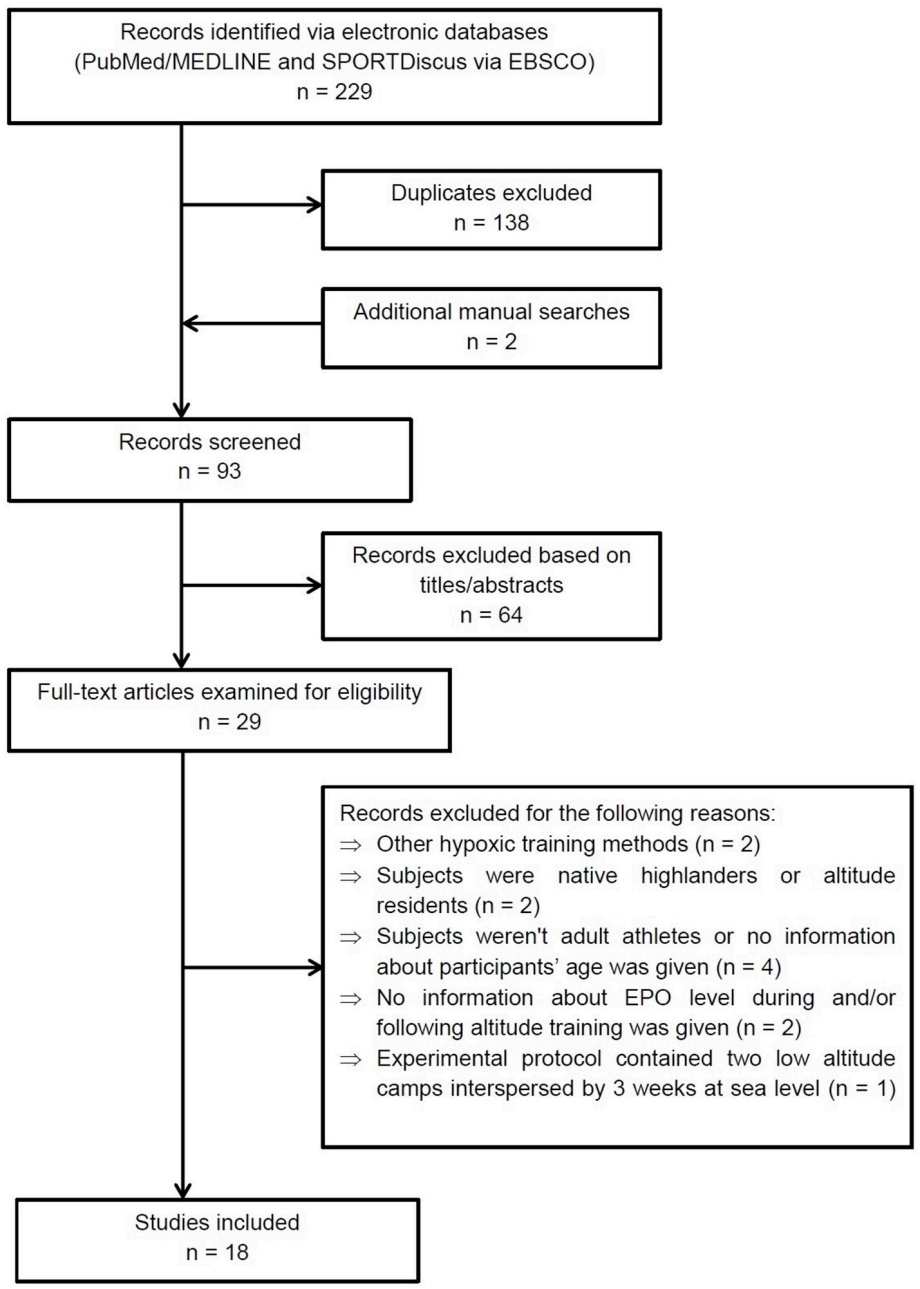
PRISMA flow diagram showing the methodology for literature review and selection of studies.

Studies included in this review met the following criteria: research was conducted on adult athletes, publications without information on the participants' age were rejected, EPO level was measured during and/or following altitude training, hematological variables were recorded before and after altitude training, study protocols concerned moderate normobaric or natural hypobaric hypoxia, altitude training was performed according to the LH-TH or LH-TL method or live high-base train high-interval train low (HiHiLo) protocol. After review of the titles, abstracts and full text, a total of 18 articles were selected for the review (see Figure 1 for details). There are many data from altitude training concerning changes of total hemoglobin mass (tHb_mass_) and other hematological variables without simultaneous EPO measurements. Therefore, we selected only some of these data to quote them in the discussion section. However, it is worth pointing out that the above-mentioned data have been comprehensively reviewed and discussed recently by Gore et al. (2013), Millet et al. (2017), and Lobigs et al. (2018).

## Results

### EPO kinetics during and after altitude training

Due to different time and frequency of blood sampling by different researchers during and after altitude training, complete unification of results from studies on EPO kinetics is difficult, but the data presented in the reviewed literature (see Table 1) allowed us to detect certain trends. During altitude training, the EPO level increases significantly after the first to third days/nights at altitude (Asano et al., 1998; Friedmann et al., 1999; Ashenden et al., 2000; Stray-Gundersen et al., 2001; Dehnert et al., 2002; Heinicke et al., 2005; Wehrlin et al., 2006; Schuler et al., 2007; Clark et al., 2009; Garvican et al., 2012a; Neya et al., 2013; Chapman et al., 2014; Czuba et al., 2014). After the peak, the EPO level start to fall gradually, but stays above initial values for a few days to weeks. Changes of EPO level become non-significant compared to baseline values after the first (Schuler et al., 2007; Garvican et al., 2012a; Chapman et al., 2014), second (Stray-Gundersen et al., 2001; Dehnert et al., 2002), or third week (Wehrlin et al., 2006; Czuba et al., 2014) at altitude.

**Table 1:** Changes in erythropoietin concentration and values of hematological variables during altitude training and after return to sea level in athletes.

**Authors**	**Type of hypoxia**	**Athletes**	**Altitude (m a.s.l.)**	**Days/nights in hypoxia**	**Daily hypoxic dose (h/day)**	**Total hours in hypoxia (h)**	**EPO concentration**		Changes of hematological variables following hypoxic training^*^
							At altitude^*^	After return to sea level^*^	
**LIVE HIGH—TRAIN LOW (LH-TL)**									
Ashenden et al., 2000	N	Runners	2,650	3 × 5	8–11	120–165	↑ after 1st and 5th night	= (2nd day)	= %Ret, [Hb] (2nd and 13th day)
Clark et al., 2009	N	Cyclists	3,000	21	14	294	↑ after 1st and 2nd night	↓-26% (2nd day) = (6th day)	↑ tHb_mass_ (3.3%) (im. and 7th day)
Dehnert et al., 2002	H	Triathletes	1,956	14	13	182	↑ after 1st and 7th night = 14th night	–	= [Hb], tHb_mass_, Hct, RBC, RCV, %Ret (im.)
Mounier et al., 2006	N	Swimmers	2,500–3,000	13	16	208	–	↓-27% (im.)	= Hct, [Hb], ↑ RCV (8.5%) (im.)
Neya et al., 2013	N	Runners	3,000	21	10	210	↑ after 1st night	-	↑ tHb_mass_ (3.5%) (7th day)
Pottgiesser et al., 2012	N	Cyclists	3,000	26	17	442	= after 7th day ↓ after 14th day	↓ (2nd and 5th day)	↑ tHb_mass_ (5,5%) (im.) = tHb_mass_ (9th day) = [Hb], Hct, % Ret (im., 2nd and 5th day)
Robach et al., 2006	N	Biathletes, Nordic-combined skiers and cross-country skiers	2,500, 3,000, 3,500	18 (3 × 6)	11	198	= after 6th day ↑ after 12th day = after 18th day	= 14th day	= RBC, RCV, [Hb], tHb_mass_, Hct, %Ret (14th day)
Schuler et al., 2007	H	Cyclists	2,340	21	19	399	↑ after 1st day = 7, 14, 21st day	-	↑ [Hb], Hct (im)
**LIVE HIGH—BASE TRAIN HIGH—INTERVAL TRAIN LOW (HiHiLo)**									
Chapman et al., 2014	H	Runners	178	28	≤24	>600	↑ after 1st and 2nd day = 3rd, 7th, 14th, 21st day	↓ (im.)	↑ RCV (~7%) (im.) = RCV (14th day)
			2,085, 2,454, 2,800				↑ after 1st, 2nd, and 3rd day = 7, 14th, 21st day	↓ (im.)	↑ RCV (~6%) (im.) = RCV (14th day)
Czuba et al., 2014	H	Biathletes	2,015	21	≤24	470-480	↑ after 1st, 3rd, 7th, and 14th day = 21st day	= (7th day)	↑ RBC (5%), [Hb] (6.4%), Hct (4.6%), %Ret (16.6%) (7th day)
Stray-Gundersen et al., 2001	H	Runners	2,500	27	≤24	>600	↑ after 1st night = 19th day	↓ - 13% (im.)	↑ [Hb] (7.5%), ↑ Hct (4.4%) (im.)
Wehrlin et al., 2006	H	Orienteering athletes	2,456	24	≤24	>500	↑ after 1st and 12th day = 24th day	= (8th day)	↑ %Ret (43%), ↑ tHb_mass_ (5.3%), ↑ RCV (5%) (8th day)
**LIVE HIGH—TRAIN HIGH (LH-TH)**									
Asano et al., 1998	H	Swimmers	1,886	21	24	504	↑ after 3rd day = 10th, 15th, 20th day	= (3th, 16th, 30st day)	= RBC, [Hb], Hct (3th, 16th, 30st day)
Chen et al., 2014	H	Long-distance track and field athletes	2,200	14	24	336	–	↑ 48% (im.)	↑[Hb], Hct, RBC (im.)
Friedmann et al., 1999	H	Boxers	1,800	18	24	432	↑ after 1st day = after 7th day ↑ after 18th day	= (5–6th day)	= RCV; % Ret; tHb_mass_ ↑ [Hb] ↓ Hct (5– 6th day)
Garvican et al., 2012a	H	Cyclists	2,760	21	24	504	↑ after 2nd night = 6th, 12th, and 21st day	↓ - 41% (im.) ↓ - 23% (10th day)	↑ tHb_mass_ (2%) (im. and 10th) = %Ret (im.) ↓ %Ret (10th day)
Heinicke et al., 2005	H	Biathletes	2,050	21	24	504	↑ after 1st, 2nd, 4th, 10th, and 20th day	= (16th day)	= tHb_mass_, Hct, RCV (16 days post)
Nadarajan et al., 2010	H	Cyclists	1,905	21	24	504	–	= (im.) ↓ (9 and 16th day)	↑ %Ret (im.) = Hct (im.) = %Ret (9 and 16th day)

*N, normobaric hypoxia; H, natural hypobaric hypoxia*;

* *Significant differences compared to baseline; = no changes; ↑ increase; ↓ decrease; im., immediately after altitude training; [Hb], hemoglobin concentration; tHb_mass_, total hemoglobin mass; %Re, percent of reticulocytes; RCV, red cell volume; RBC, red blood cell count; Hct, hematocrit value; m a.s.l, meters above sea level*.

Following a few weeks of altitude training, after returning to sea level, EPO concentration returns to baseline immediately or after a few days (Asano et al., 1998; Friedmann et al., 1999; Ashenden et al., 2000; Wehrlin et al., 2006; Czuba et al., 2014) or even declines below the initial value (Stray-Gundersen et al., 2001; Mounier et al., 2006; Clark et al., 2009; Garvican et al., 2012a; Chapman et al., 2014).

### Changes of hematological variables following altitude training

Some of the studies show that LH-TH, LH-TL, and HiHiLo protocols all have beneficial effects on the hematological variables (Table 1). Following altitude training, RCV (Mounier et al., 2006; Wehrlin et al., 2006; Chapman et al., 2014) and tHb_mass_ increase relative to the pre-altitude values (Wehrlin et al., 2006; Clark et al., 2009; Garvican et al., 2012a; Pottgiesser et al., 2012; Neya et al., 2013). Also hemoglobin concentration ([Hb]), hematocrit value (Hct), red blood cell count (RBC), and percent of reticulocytes (%Ret) are elevated after altitude training (Stray-Gundersen et al., 2001; Wehrlin et al., 2006; Schuler et al., 2007; Nadarajan et al., 2010; Chen et al., 2014; Czuba et al., 2014).

However, there are studies in which hematological variables do not improve after altitude training. The results obtained by Friedmann et al. (1999); Dehnert et al. (2002); Heinicke et al. (2005), and Robach et al. (2006) indicated that tHb_mass_ and RCV did not increase after the LH-TH or LH-TL protocol. In some studies also other hematological variables did not change significantly (Asano et al., 1998; Ashenden et al., 2000; Dehnert et al., 2002; Heinicke et al., 2005; Robach et al., 2006).

It also happened that even if a significant increase in EPO level during altitude training was observed, after return to sea level, improvement of hematological variables was not observed (Asano et al., 1998; Ashenden et al., 2000; Dehnert et al., 2002; Heinicke et al., 2005; Robach et al., 2006).

## Discussion

### EPO response to altitude training

**Figure 2:**
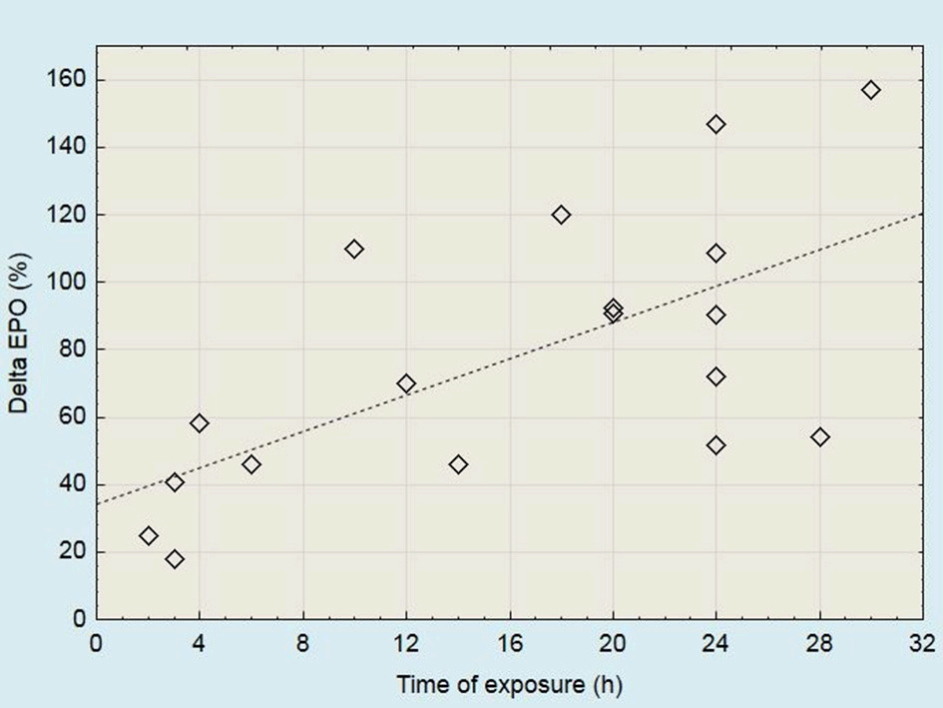
Acute changes in serum EPO levels following the exposure to moderate altitude (2,000 to 3,100 m). Data derived from studies published by Chapman et al. (1998, 2010, 2014); Stray-Gundersen et al. (2001); Jedlickova et al. (2003)^*^; Friedmann et al. (2005)^*^; González et al. (2006)^*^; Mounier et al. (2006); Wehrlin et al. (2006); Mackenzie et al. (2008); Clark et al. (2009)^*^; Neya et al. (2013); Badenhorst et al. (2014); Czuba et al. (2014). ^*^More than one measuring point has been presented in these papers.

Studies have shown that EPO level increases following several dozen minutes or several hours of acute hypoxic exposure (Eckardt et al., 1989; Knaupp et al., 1992; Rodríguez et al., 2000; Mackenzie et al., 2008). The increase in the EPO level is higher when there is a greater decline in partial pressure of oxygen in arterial blood (PaO_2_) (Eckardt et al., 1989). Acute changes in EPO level following exposure to moderate altitude are presented in Figure 2. During continuous exposure to altitude, EPO reached a peak after 1–3 days (Berglund et al., 1992; Jelkmann, 2011). This observation is confirmed by the results of the studies presented in Table 1.

In subsequent days of staying at altitude, after reaching the peak, the EPO level starts to fall gradually. The rate of decline may vary, but it seems that it does not depend on the altitude training protocol which was used. A factor that may affect the rate of decline of EPO is the hypoxic level. With an altitude below the threshold of ~2,000 m, even if it is sufficient to stimulate EPO production, the EPO level may start to drop rapidly. Li et al. (2002) observed a significant increase in EPO levels following 6 h of hypoxia at different altitudes (1,780–2,800 m). After 24 h, the EPO level continued to increase, but only for 2,454 and 2,800 m, whereas at altitudes 1,780 and 2,085 m, EPO started to decline. A similar comparison was made by Chapman et al. (2014), who documented a rise in EPO levels after 24 h at altitude, but after 72 h in the 1,780 m group, EPO returned to baseline levels, while it remained elevated in those individuals staying at a higher altitude.

### Causes of the reduction in EPO level

The mechanisms of the reduction in EPO levels during altitude training are still unclear. On consecutive days staying at altitude, a gradual fall of EPO level with an opposite tendency of peripheral oxygen saturation (SpO_2_) changes is observed (Ashenden et al., 2000; Czuba et al., 2018). Furthermore, the kinetics of EPO level in response to hypoxia are similar to hypoxia-inducible factor 1 (HIF-1) protein level kinetics. Lundby et al. (2009) pointed out that in response to hypoxic exposure, the HIF-1 protein level peaks within the first hours and gradually falls toward the initial values. Therefore, a progressive decline of EPO level and the degree of this decline are probably associated with the phenomenon of stabilization of HIF-1α. These data show that progressive acclimatization may be one of the causes of reduced EPO production in successive days and weeks during altitude training.

On the other hand, it is possible that production of EPO is not inhibited. Rusko et al. (2004) observed that serum EPO levels reflected a balance between renal EPO production and its consumption in the bone marrow. In the first hours at altitude, EPO production is higher than its consumption. The decline in EPO concentration results from elevated erythropoiesis that leads to greater EPO consumption during chronic altitude exposure. The increase of reticulocyte production accompanying the EPO decrease at altitude (Mairbäurl, 1994; Stray-Gundersen et al., 2001; González et al., 2006) supports this argument.

### Hematological response to altitude training

One of the most expected benefits of chronic hypoxia is to improve hematological variables. Exposure to a terrestrial or simulated hypoxic environment leads to a reduction in the PaO_2_ as well as in arterial oxygen saturation (SaO_2_) (Rusko et al., 2004). Data obtained by Eckardt et al. (1989) indicate that a higher hypoxic level causes a greater decline of PaO_2_. The decrease in PaO_2_ stimulates EPO production (Jelkmann, 2011). Consequently, the exposure to hypoxia induces an increase in EPO production by the kidneys, which in turn accelerates erythropoiesis. This mechanism has not been completely explored, but it is claimed that HIF-1α is the major and pivotal mediator of this erythropoietic cascade (Caro, 2001; Prabhakar, 2001; Wilber et al., 2007). The physiological threshold for acceleration of erythropoiesis has been described to be at PaO_2_ levels of ~70 mmHg, which corresponds to exposure to an altitude of circa 1,600 m (Weil et al., 1968). However, Chapman et al. (1998) reported individual variability in the altitude-induced erythropoietic response ranging from 8.0 to 37.5 mU/ml at 2,500 m, with further dependency upon exposure time.

A rise in erythropoiesis is possible if the erythropoietic cascade is started. Previous studies have shown that the increase of the EPO level and acceleration of erythropoiesis depend on the “hypoxic dose.” Both level and duration of hypoxic exposure can influence the hematological response to altitude training. The results of the meta-analysis carried out by Gore et al. (2013) indicated that tHb_mass_ increases at ~1.1%/100 h of altitude for LH-TL. The anticipated rise of tHb_mass_ was ~3.4% after 2 weeks of LH-TH training (>2100 m). The LH-TL protocol is just as effective as LH-TH when the total hours of hypoxia are matched, and altitude increases to ~3,000 m. Likewise, Rusko et al. (2004) suggested that the minimum time to reach an acclimatization effect and improve blood oxygen-carrying capacity appears to be >12 h per day for at least 3 weeks at an altitude of 2,100 to 2,500 m. Similarly, data obtained by Li et al. (2002) showed that altitudes of >2,100 to 2,500 m are needed to stimulate EPO production. Wilber et al. (2007) argued that the altitude of 2,000 to 2,500 m is sufficient if the daily dose of hypoxic exposure is higher than 22 h for a minimum of 4 weeks. Fewer hours (12–16 h) may be also used, but exposure to a higher altitude (2,500 to 3,000 m) is necessary to reproduce the erythropoietic effects.

The above data show that the hematological response may not be triggered when the altitude is too low or when the total time of hypoxic exposure is too short. Studies presented in Table 1 indicate that when the total duration of hypoxic exposure was <200 h (Ashenden et al., 2000; Dehnert et al., 2002; Robach et al., 2006), a hematological response was not elicited. In turn, other research indicate that total time in hypoxia of about 210 h was sufficient to increase RCV (Mounier et al., 2006) or tHb_mass_ (Neya et al., 2013). However, in these studies the altitude to which the athletes were exposed was higher, reaching ~3,000 m. The results of some studies (Asano et al., 1998; Dehnert et al., 2002) also show that an altitude <2,000 m may be ineffective for improving hematological variables. However, the data presented by Chapman et al. (2014) and Nadarajan et al. (2010) indicate that it is possible to improve RCV or %Ret after training performed at an altitude below 2,000 m. Other studies support these observations. Garvican-Lewis et al. (2015) indicated that after 3-week training at 1,800 m, tHb_mass_ increased by 3% and it was higher than in the control group, which trained at 600 m. Furthermore, Frese and Friedmann-Bette (2010) carried out an experiment in which two training camps were held at altitudes of 1,300 and 1,650 m (LH-TH), separated by training at sea level. They reported a non-significant increase in tHb_mass_ after the first 3 weeks of altitude training, but after the second training camp tHb_mass_ increased by 5.1% compared to the pre-test values. In this experiment, the cumulative time at altitude was ~1,000 h and perhaps compensated for the lower altitude to elicit a hematological response.

These observations indicate that the total dose of hypoxia resulting from the combination of altitude and time of exposure seems to determine the hematological response to altitude training. As Garvican-Lewis et al. (2015) point out, further research is needed to better understand the relationship between hypoxic dose and improvement of hematological variables, and to determine the minimally effective altitude.

### Optimization of different hypoxic doses

Attempts to optimize different doses of altitude exposure are still underway. This optimization would make it possible to establish a minimum threshold efficacy of hypoxic exposure. Garvican-Lewis et al. (2016) proposed a new metric, with hypoxic dose termed “kilometer hours” and defined as km·h (“km” denotes elevation of the exposure and “h” is the total duration of the exposure). This model integrates the role of exposure time and altitude. However, Millet et al. (2016) stressed the large interindividual variability in the physiological responses to a specific hypoxic dose; therefore, they proposed a metric based on the magnitude of the stimulus rather than on the altitude. This metric has been termed saturation hours and defined as %·h = (98/s - 1) x h x 100, where “s” is the saturation value (in %) and “h” is the time (in hours) sustained at any second level.

As Millet et al. (2016) rightly pointed, the metric proposed by Garvican-Lewis et al. (2016) concerns the hypoxic stimulus, while ”saturation hours“ refers to the hypoxic response. Such an approach could help to individualize training. For coaching practice, the use of the “saturation hours” metric means that to obtain the same “hypoxic dose,” those athletes with a higher saturation value or a faster rise in SpO_2_ during altitude training should extend their training time or increase exposure to a higher altitude to reduce saturation. This approach would increase the efficacy of altitude/hypoxic training by considering the individual response to applied stimuli.

Undoubtedly, standardization of hypoxic dose metrics would help establish clear guidelines for altitude training prescription, which includes the potential for a targeted approach based on individual response patterns, whilst providing a stronger framework for interpreting and comparing different training protocols employed in research and practice.

### Maintenance of post-altitude training effects

The positive effects of altitude training in athletes are temporary (Wehrlin et al., 2016). Even if immediately after or a few days after a return to sea level, hematological variables are elevated, some data indicate that nearly all of the hypoxia-induced changes may be lost within 1–2 weeks (Pottgiesser et al., 2012). Chapman et al. (2014) noted a decrease in RCV, while Brugniaux et al. (2006) observed a drop in tHb_mass_ to the baseline level after 14 to 15 days following the completion of altitude training. This loss of effect may be why some studies did not detect improvements in hematological variables despite applying the recommended hypoxic dose. When the only post-altitude measurement is made after 14 days, the effects may not be observed (Heinicke et al., 2005; Robach et al., 2006). On the other hand, in a study conducted by Brocherie et al. (2015), the tHb_mass_ was significantly higher 3 weeks after altitude training compared to pre-altitude levels and this result remains consistent with the model proposed by Gore et al. (2013), where gains in tHb_mass_ values are estimated for ~3 weeks.

Interestingly, even if hypoxia promotes erythropoiesis, it remains uncertain whether this will lead to an increase in RBC, as in certain conditions the reticulocytes may be targeted for early destruction (Hahn and Gore, 2001). This is likely linked to the phenomenon of neocytolysis. Neocytolysis is a physiological process that controls red cell mass by down-regulation if it is excessive. Because the absence of hypoxic stress after return to sea level causes a decline of the EPO level, neocytolysis is accelerated, which consequently leads to selective hemolysis of the youngest circulating red blood cells (Alfrey et al., 1997; Rice et al., 2001; Rice and Alfrey, 2005). A significant decrease in tHb_mass_, fall of erythrocyte survival time, lower iron turnover and reduction of bone marrow production of erythroid cell lines are observed after the return to sea level of both native highlanders and those individuals who stayed at altitude temporarily (Levine, 2002; Prommer et al., 2010). These results suggest that neocytolysis may be one of the causes of the lack of improvement in hematological variables after return to sea level even if altitude training favored erythropoiesis.

For athletes, the duration of maintaining the post-altitude training effects is a very important issue, because it determines the moment of the return to sea level before the competition. Schmidt and Prommer (2008) noted that tHb_mass_ is reduced again by 50% after 3 weeks following the return to sea level. Therefore, the time between altitude training and the competition should be <20 days. Dick (1992) claimed that the maximum performance occurs between 15 and 24–28 days, whereas the first week following altitude training is characterized by poor performance. Conversely, Heinicke et al. (2005) found that the re-acclimatization period prior to competition should be very short (1–3 days). Chapman et al. (2014) concluded that interaction between hematological and other physiological parameters influences the optimal time to compete after altitude training. Furthermore, Turner et al. (2017), suggested that additional normobaric hypoxic exposure (4,200 m and above, 2 h per day) after an altitude training camp can prevent a sudden decline in EPO levels and help maintain an improved tHb_mass_ level. This problem is yet to be explained, and practical application should be individually adapted to athletes.

### Methodological aspects

Based on the majority of the reviewed literature, it would seem logical to suppose that the conflicting and inconsistent reports concerning tHb_mass_ after altitude training might be, at least partially, attributed to different methodological approaches used by researchers. It was indicated that not all techniques had the same precision and were suitable for the research concerning training in hypoxia (Wehrlin et al., 2016). Furthermore, Garvican et al. (2012b) and Millet et al. (2017) provided evidence that the use of some tHb_mass_ measurements may lead to either overestimated or underestimated tHb_mass_ values, which makes it difficult to achieve unequivocal interpretation of the results and evaluation of the actual effects of altitude training. Based on the comments by the above mentioned authors, one may suppose that among currently available techniques the most precise method for tHb_mass_ estimation is the CO-rebreathing method with refinements that allow for minimization of measurement errors and determination of individual responses to altitude/normobaric hypoxia exposure (Garvican et al., 2012b).

### Individual variability of EPO production and hematological adaptations

The rate of EPO production is related to the level of hypoxic stress, but the increase in EPO induced by altitude is characterized by inter-individual variability. The difference of EPO level after exposure to the same altitude can be up to several hundred percent between subjects (Table 2). The study conducted by Li et al. (2002) showed that some subjects achieved about a 100% increase in EPO levels after exposure to 1,780 m, whereas others did not respond to the altitude of 2,805 m. The individual EPO response may affect the level of hematological adaptation and performance in athletes after altitude training. Chapman et al. (1998) collected data from 39 athletes living at an altitude of 2,500 m. The athletes performed a 4-week training block at altitudes between 1,200 and 3,000 m. Based on the improvements in sea-level performance during a 5,000 m run, the study group was retrospectively divided into responders and non-responders to altitude training. It was found that the responders had significantly larger increases in mean EPO concentration after 30 h of hypoxia compared to the non-responders. After 14 days of exposure to altitude, EPO remained elevated only in responders. It is likely that for non-responders, greater hypoxic stimuli may be needed to induce a sufficiently large release in EPO and augment red cell production (Chapman et al., 1998). These observations showed that individual aptitudes of athletes play an important role in the efficiency of altitude/hypoxic training.

**Table 2:** Individual variability of EPO levels after exposure to moderate altitude.

**Authors**	**Number of participants**	**Exposure time (h)**	**Altitude (m a.s.l.)**	**ΔEPO (compared to initial values)**
Jedlickova et al., 2003	48	24	2,800	−41 to +433%
Chapman et al., 2010	26	20	2,500	−20 to +415%
González et al., 2006	63	12	2,200	−14 to +317%
Friedmann et al., 2005	16	4	2,500	+10 to +185%
Mackenzie et al., 2008	10	2	3,100	−5 to +62%

*m a.s.l., meters above sea level*.

Individual variability in response to altitude training is also important in the context of the efficiency of anti-doping tools such as the Athlete Biological Passport (ABP). The results of a meta-analysis conducted by Lobigs et al. (2018) indicated that due to the complex nature of the response to altitude training, altitude still cannot be directly included in the ABP algorithm. However, better knowledge of the effects of altitude training on blood markers can make it easier for experts to make a judgment on a dubious ABP profile. We believe that the information presented in our review may also be a guide for experts concerning the impact of altitude training on erythropoietic response and hematological variables.

### Genetic determinants of the hypoxic response

Various mechanisms have so far been suggested as responsible for individual variability, but many factors associated with the altitude response seem to be genetically determined (Chapman et al., 1998; Li et al., 2002). Attempts to find genetic determinants of the hypoxic response are in progress.

For instance, Witkowski et al. (2002) suggested that a specific haplotype of the EPO gene may exist, which can be useful in predicting the erythropoietic response to altitude. Further analysis (Jedlickova et al., 2003) provided more information on this issue. In this study, based on the level of increase in the EPO level after hypoxic exposure, athletes were divided into low responders, intermediate responders, and high responders. The obtained data demonstrated that the 185-bp allele of the EPO gene was most prevalent in the group of high responders. The 185-bp allele showed a significant correlation with the EPO hypoxic response. When all study participants were considered, the increase in EPO in the group of people with the 185-bp allele was 135%, whereas in the group without this allele, the EPO level rose by 78%.

In other studies, the association between polymorphism of the HIF-1α gene and hypoxic response was analyzed. Tanimoto et al. (2003) demonstrated that the T allele (Ser582) compared to the C allele (Pro582) is related to higher HIF-1α activity. In turn, Liu and Hu (2006) demonstrated a higher SaO_2_ during exercise performed in hypoxia in subjects with the CT genotype. Furthermore, following 4 weeks of hypoxic exposure and hypoxic training, changes in maximal oxygen uptake (VO_2_max) were higher for the CT genotype compared to the CC genotype.

However, there are data that do not confirm the above observations. Hennis et al. (2010) examined whether the presence of the T allele impacts the EPO level, hypoxic ventilatory response (HVR) and SaO_2_. However, the results demonstrated that the CT/TT genotype was not associated with an improved response for 8 h hypoxic exposure. No differences were observed in the increase in EPO levels following 4 and 8 h of exposure or in the levels of SaO_2_ and HVR between the CC genotype and CT/TT genotypes. Also, Richalet et al. (2009) did not detect a correlation between HIF-1α genotypes and HVR during exercise in normobaric hypoxia.

The potential relationship between the ACE gene polymorphism (I/D) and hypoxic response was also analyzed. Angiotensin II is involved in the regulation of EPO production and it can increase EPO level (Freudenthaler et al., 2000; Gossmann et al., 2001). Presence of the I allele of the ACE gene reduces enzymatic activity and, consequently, decreases the level of angiotensin II. This suggests that the DD genotype may be associated with a greater increase in EPO level after hypoxic exposure. However, González et al. (2006) reported no significant differences in EPO levels between the DD and ID/II genotypes. The responders and non-responders had similar genotypic profiles. These results led to the conclusion that polymorphism of the ACE gene does not influence the EPO response in athletes during exposure to hypoxia.

It turns out that the I allele of the ACE gene is identified in mountaineers more often than the D allele (Martin et al., 2010). Woods et al. (2002) found that presence of the I allele can produce benefits at high altitude due to prolonged maintenance of SaO_2_. Furthermore, Bigham et al. (2008) observed higher values of SaO_2_ both at rest and during exercise at high altitudes (~4,000 m) in subjects with the I/I genotype compared to other genotypes. However, other research, conducted by Patel et al. (2003) and Richalet et al. (2009), revealed no differences between ACE genotypes in the values of HVR and SaO_2_ during exercise in hypoxia.

Current reports on genetic determinants of the hypoxic response are few and ambiguous. Therefore, further research is needed, because such data may be useful in predicting the response to altitude training.

### Other factors affecting hematological responses to altitude training

#### Body iron stores

In addition to an insufficient hypoxic dose, the effectiveness of altitude training may be disturbed by other factors. One of them is insufficient body iron stores. At high altitude, when erythropoiesis is activated, the iron demand increases. Erythropoiesis stimulation leads to reduced production of hepcidin, a peptide hormone regulating body iron homeostasis (Robach et al., 2009; Badenhorst et al., 2014). These changes allow for enhanced iron absorption and release of iron stores (Goetze et al., 2013). The reduced serum ferritin levels observed at altitude in several studies (Roberts and Smith, 1992; Stray-Gundersen et al., 2001; Heinicke et al., 2005; Goetze et al., 2013) reflect the enhanced erythropoietic activity and increased iron turnover. However, if iron stores are insufficient or a diet at altitude is inadequate for the nutritional demands (Michalczyk et al., 2016), a hematological response will not be elicited. This interpretation is consistent with data obtained by Stray-Gundersen et al. (1992), who showed that pre-altitude iron deficiency (serum ferritin <30 ng/ml in men and <20 ng/ml in women) led to no improvements in RCV in responses to altitude exposure. Therefore, ferritin levels must be monitored on a regular basis before and during the hypoxic exposure if the aim of altitude training is to improve blood oxygen-carrying capacity. It is also recommended to supplement iron in athletes during altitude training to prevent anemia from occurring (Michalczyk et al., 2016) and to support tHb_mass_ production (Govus et al., 2015). Supplementation, depending on the pre-altitude level of serum ferritin, should be from around 40 mg to above 200 mg of elemental iron/day (Constantini et al., 2017).

#### Anabolic-catabolic balance

The hematological response to altitude training may also be limited by inadequate choice of training loads. Type of training and choice of training loads affect the direction and scope of hormonal changes (Lehmann et al., 1992; Fry and Kraemer, 1997). Testosterone (T) and cortisol (C) are steroid hormones that regulate anabolic and catabolic changes in the body. The testosterone to cortisol ratio (T/C) can be used to assess the anabolic-catabolic balance (Chicharro et al., 1998). The significant reduction of the T/C ratio during altitude training can lower the rate of erythropoiesis, especially in the early phase of acclimatization (Berglund, 1992). Although there are reports in which an increase in resting cortisol (Vasankari et al., 1993; Wilber et al., 2000) and decrease in resting testosterone (Gore et al., 1998) were observed in athletes following altitude training, it seems that these changes may have been caused by an increased training load rather than hypoxic stress (Wilber et al., 2000). Based on the above reports, it can be concluded that the correct choice of training loads and achievement of a favorable T/C ratio are additional important factors affecting the rate of erythropoiesis and the effectiveness of altitude training.

#### Injury or infection

Another factor which is likely to impair the erythropoietic response is injury or infection (McLean et al., 2013; Wachsmuth et al., 2013), because the EPO rise may be limited by inflammatory cytokines (Jelkmann et al., 1990; Fandrey and Jelkmann, 1991). However, in the most recent study, Turner et al. (2017) showed that baseline level of interleukin-6 (IL-6), and tumor necrosis factor alpha (TNFα) were not correlated with the percentage difference of the peak in EPO during hypoxic exposure to 3,600–4,800 m, which means that a significant increase of EPO level was possible even at high levels of IL-6 and TNFα. In view of the ambiguous results of previous studies, regulation of EPO production by inflammatory cytokines and the relationship of injury and infection with the response to hypoxia remain unclear and require further research.

#### Initial tHb_mass_ level

Data on the impact of initial tHb_mass_ level on improvement of tHb_mass_ after altitude training are still inconclusive. It is likely that a physiological limit of the total circulating quantity of tHb_mass_ exists. Elite endurance athletes, in response to many years of sea level training, may have maximized their tHb_mass_ level. Therefore, they might have a limited scope for increases in hematological variables following altitude training (Gore et al., 1998; Robach and Lundby, 2012; Lobigs et al., 2018). However, recent data presented by Hauser et al. (2017) indicate that absolute initial tHb_mass_ had no effect on improvement of tHb_mass_ after LH-TL. Therefore, it seems that even elite athletes with higher initial tHb_mass_ can expect tHb_mass_ improvement after LH-TL. This observation is supported by findings presented in studies by Millet et al. (2017), who demonstrated that the initial levels of tHb_mass_ were not significantly related to the increase of tHb_mass_ following LH-TL training, even in athletes with tHb_mass_ >14 g/kg.

#### Type of hypoxia

Another question concerns the possibility of different physiological responses between hypobaric and normobaric hypoxia. Opinions are still divided (Millet et al., 2012; Mounier and Brugniaux, 2012). Interesting results were obtained by Saugy and colleagues during a two-stage project. The first stage (Saugy et al., 2014) showed that the RBC, [Hb] and Hct were higher in the hypobaric group than in the normobaric group following an 18-day LH-TL camp. The same experiment was repeated, but using a crossover study and reduction of the possible group effect, and indicated that the post-LH-TL hematological responses and performance improvements were similar for hypobaric and normobaric stimuli (Saugy et al., 2016). However, the small number of previous studies comparing hypobaric and normobaric hypoxia does not allow us to state clearly whether the type of hypoxia significantly affects the difference in hematological responses after altitude training.

#### Hemoconcentration

It should be noted that the blood volume (BV) adjustments during acclimatization to altitude have two phases. In the early phase, which begins within hours of altitude exposure and lasts for the first 3–4 weeks, plasma volume (PV) decreases, causing hemoconcentration. One of the mechanisms leading to a reduction in PV at altitude is dehydration associated with increased respiratory water loss due to enhanced ventilation and increased urinary water loss observed at altitudes (Sawka et al., 2000; Michalczyk et al., 2016). In this first phase of acclimatization, PV is decreased while erythrocyte volume remains stable, resulting in increased [Hb] and reduced BV. During the second phase of acclimatization, as a result of continuous exposure to altitude for several weeks or months, erythrocyte volume increases. This phase can be accelerated by an intensive program of aerobic training at high altitudes (Sawka et al., 2000).

Changes in BV during and after hypoxic exposure can cause errors in interpretation of research results and evaluation of the actual effects of altitude training may be impeded. Hemoconcentration may explain the increase in hematological variables, especially in the early days of altitude training. It is also likely that differences in EPO levels following hypoxic exposure partly result from the decrease in PV rather than from significant diversity of EPO production. Monitoring of urine specific gravity during altitude training is essential to eliminate the hydration effect on EPO values. If this condition is not met, it may turn out that the variability range of EPO level, especially after hypoxic exposure for over 12 h, may differ from the actual variability in EPO production.

### Identification of responders and non-responders

Due to the individual variability of EPO production and many factors affecting the hypoxia response, identification of responders and non-responders among athletes prior to altitude training seems to be useful in optimizing the training process.

As suggested by Chapman et al. (1998), the number of non-responders can be minimized by screening of the erythropoietic and training velocity response to acute hypoxic exposure. In studies conducted by Friedmann et al. (2005), the EPO increase following 4 h of exposure to normobaric hypoxia was significantly correlated with the acute EPO rise during altitude training (after the first and second day). These findings indicate that short hypoxic exposure can be used to identify athletes who respond to altitude training. Based on the results of previous research (Figure 2), it can be assumed that the EPO level after 24 h of exposure to moderate altitude should be at least doubled in relation to baseline. The smaller rise may suggest that the response to altitude/hypoxic training will also be weak.

However, as we observed on the basis of the reviewed studies, even a significant increase in EPO level during altitude training does not guarantee improvement of hematological variables after the return to sea level (see Table 1). This observation is in line with previous reports in which correlations were not found between the EPO levels following exposure to altitude and tHb_mass_ (Rusko et al., 1999; Friedmann et al., 2005). Therefore, it is doubtful whether the improvements of hematological variables after altitude training can be predicted using the EPO response to acute hypoxic exposure. Determining efficacious methods to identify better and worse responders prior to altitude training remains a topic for further research.

Regardless of attempts to find an effective tool for identification of responders and non-responders prior to altitude training, daily measurement of SpO_2_ at rest during altitude training may by a useful and inexpensive method to analyze the hematological response to chronic hypoxia. Millet et al. (2016) noted that the SpO_2_ could be easily measured without limitations or inconvenience to athletes through development of sportswear incorporating an oximeter inside the textile. It is known that the increase in EPO levels is directly proportional to the level of hypoxia and decline in SpO_2_ (Eckardt et al., 1989). In our recent study Czuba et al. (2018) conducted during successive weeks of altitude training, SpO_2_ in the altitude training group showed an upward tendency that was opposite to changes in EPO levels. Consequently, the effect of hypoxia on blood EPO levels became weaker with increasing acclimation, which was caused by the improvement in blood oxygen-carrying capacity. Since the desaturation level depends not only on altitude but is highly individualized and also dependent on the training background of athletes or previous altitude experiences (Chapman et al., 1998, 2014; Millet et al., 2016), the measurement of SpO_2_ during altitude training will allow for the measurement of the time the athlete has spent at a given saturation level and determine the actual magnitude of the stimulus individually for each athlete (Millet et al., 2016).

## Conclusions and practical applications

Despite the knowledge developed over the years, EPO still remains the subject of researchers' interest. As noted by Lundby (2011), new discoveries in recent years have appeared in the field of the functions and synthesis of EPO. However, in the context of altitude training, what seems to be the most important is that the elevated EPO production by the kidneys in hypoxia is a key factor enabling subsequent improvement of hematological variables. However, even a significant increase in EPO level during altitude training does not guarantee improvement of hematological variables after the return to sea level. The most recent publications reviewed show that the effectiveness of altitude training depends on many variables. Therefore, effective planning and implementation of LH-TH, LH-TL, or even HiHiLo training methods should take into consideration the following points:

- Serum EPO levels rise at altitude and, after reaching a peak, they gradually decrease to baseline levels. This decline is a physiological phenomenon resulting from progressive acclimatization and/or increased stimulation of erythropoiesis (Eckardt et al., 1990; Rusko et al., 2004; Czuba et al., 2014).
- Total hypoxic dose resulting from the combination of altitude level and exposure duration seems to determine the hematological response to altitude training. The hypoxic dose should be at least 250 h at an altitude of 2,100 to 2,500 m (Rusko et al., 2004; Chapman et al., 2014). It can be expected that the tHb_mass_ increase will be ~1.1% for every 100 h spent in hypoxia (Gore et al., 2013). A new metric for determining the hypoxic dose, which integrates the role of exposure time and altitude, is ”kilometer hours“ (Garvican-Lewis et al., 2016).
- For coaching practice, application of the “saturation hours” metric may be useful. The measurement of SpO_2_ during altitude training will allow measurement of the time the athlete has spent at a given saturation level. This approach would increase the efficacy of altitude/hypoxic training by considering the individual response to applied stimuli (Millet et al., 2016).
- Considering a fast return of tHb_mass_ level and RBC to baseline, the time between the return to sea level after altitude training and a competition should be between 2 and 3 weeks (Dick, 1992; Schmidt and Prommer, 2008) or be extremely short (1–3 days) (Heinicke et al., 2005).
- Inadequate hypoxic dose and training content, neocytolysis, insufficient body iron stores, injury and infections, disturbed anabolic-catabolic balance, or the physiological limit of tHb_mass_ are likely to lead to no improvement in hematological variables in athletes after altitude training (Stray-Gundersen et al., 1992; Gore et al., 1998; Rusko et al., 2004; Rice and Alfrey, 2005; Wachsmuth et al., 2013).
- Individual variability of EPO production can affect the altitude response (Chapman et al., 1998; Li et al., 2002). EPO production, like many other factors associated with the response to altitude, may be genetically determined, but further research is needed to identify the genetic determinants of the hypoxic response.

## Author contributions

KP, MC, and JL conceptualization and writing and editing; MC and JL supervision and funding acquisition. All authors have read and approved the final version of the manuscript. All authors made a significant contribution to this study.

### Conflict of interest statement

The authors declare that the research was conducted in the absence of any commercial or financial relationships that could be construed as a potential conflict of interest.
